# The Mu Subunit of Plasmodium falciparum Clathrin-Associated Adaptor Protein 2 Modulates *In Vitro* Parasite Response to Artemisinin and Quinine

**DOI:** 10.1128/AAC.04067-14

**Published:** 2015-04-10

**Authors:** Gisela Henriques, Donelly A. van Schalkwyk, Rebekah Burrow, David C. Warhurst, Eloise Thompson, David A. Baker, David A. Fidock, Rachel Hallett, Christian Flueck, Colin J. Sutherland

**Affiliations:** a Department of Immunology and Infection, Faculty of Infectious and Tropical Diseases, London School of Hygiene and Tropical Medicine, London, United Kingdom; b Department of Pathogen Molecular Biology, Faculty of Infectious and Tropical Diseases, London School of Hygiene and Tropical Medicine, London, United Kingdom; c Department of Microbiology and Immunology, Columbia University College of Physicians and Surgeons, New York, New York, USA; d Division of Infectious Diseases, Department of Medicine, Columbia University College of Physicians and Surgeons, New York, New York, USA; e Department of Clinical Parasitology, Hospital for Tropical Diseases, University College London Hospital, London, United Kingdom

## Abstract

The emergence of drug-resistant parasites is a serious threat faced by malaria control programs. Understanding the genetic basis of resistance is critical to the success of treatment and intervention strategies. A novel locus associated with antimalarial resistance, *ap2-mu* (encoding the mu chain of the adaptor protein 2 [AP2] complex), was recently identified in studies on the rodent malaria parasite Plasmodium chabaudi (*pcap2-mu*). Furthermore, analysis in Kenyan malaria patients of polymorphisms in the Plasmodium falciparum *ap2-mu* homologue, *pfap2-mu*, found evidence that differences in the amino acid encoded by codon 160 are associated with enhanced parasite survival *in vivo* following combination treatments which included artemisinin derivatives. Here, we characterize the role of *pfap2-mu* in mediating the *in vitro* antimalarial drug response of P. falciparum by generating transgenic parasites constitutively expressing codon 160 encoding either the wild-type Ser (Ser160) or the Asn mutant (160Asn) form of *pfap2-mu*. Transgenic parasites carrying the *pfap2-mu* 160Asn allele were significantly less sensitive to dihydroartemisinin using a standard 48-h *in vitro* test, providing direct evidence of an altered parasite response to artemisinin. Our data also provide evidence that *pfap2-mu* variants can modulate parasite sensitivity to quinine. No evidence was found that *pfap2-mu* variants contribute to the slow-clearance phenotype exhibited by P. falciparum in Cambodian patients treated with artesunate monotherapy. These findings provide compelling evidence that *pfap2-mu* can modulate P. falciparum responses to multiple drugs. We propose that this gene should be evaluated further as a potential molecular marker of antimalarial resistance.

## INTRODUCTION

Antimalarial drugs remain indispensable tools in the fight against malaria. The potent artemisinin derivatives, combined with longer-half-life partner drugs, are the only efficacious treatments left for multidrug-resistant Plasmodium falciparum infection and thus form the cornerstone of antimalarial drug therapy. The emergence of drug resistance represents one of the most serious problems faced by malaria control programs. Historically, parasite resistance to antimalarial medicines emerged in Southeast Asia and eventually spread toward Africa, and, alarmingly, there is already evidence of reduced susceptibility of P. falciparum to artemisinin derivatives in Southeast Asia, as evidenced by delayed parasite clearance times *in vivo* (1–4). For now, the delayed-clearance phenotype appears to be confined to the Greater Mekong subregion (5); however, this reduced artemisinin sensitivity may spread to other regions or independently arise elsewhere, including Africa, where the burden of malaria is highest and where the emergence of resistance would have a terrible impact. A better understanding of the mechanisms of artemisinin resistance would be a major advance in our ability to develop and validate new tools for resistance surveillance. These are essential tools to guide national treatment policies and help the design and deployment of new drug combinations that may deter the emergence and spread of resistance.

Mutations within the P. falciparum K13 kelch propeller domain gene (*pfk13*) recently identified in Cambodian parasite populations have been proposed as a molecular marker of artemisinin resistance (6). Other genes acting together with *pfk13* in Cambodia may be involved in the slow-clearance phenotype *in vivo*, and K13-independent mechanisms may have arisen in other settings. Other potential genetic markers of artemisinin resistance have previously been identified using genome-wide sequencing of drug-pressured mutants of the rodent malaria parasite Plasmodium chabaudi (7). One of these, the gene encoding the mu subunit of the adaptor protein 2 (AP2) complex involved in clathrin-mediated endocytosis, exhibits polymorphism in P. falciparum isolates from Africa (8).

We recently reported a phenomenon of submicroscopic persistent parasites at day 3 following treatment with artemisinin combination therapy (ACT) in Kenyan children. These persistent parasites were detectable only by quantitative PCR (qPCR), but the children carrying these parasites had a higher mosquito transmission potential and were more likely to go on to classical treatment failure at day 28 or 42 posttreatment (9). Sequence analysis of these parasites revealed a particular genotype, combining variants of the *pfcrt*, *pfmdr1*, *pfap2-mu*, and *pfubp1* candidate drug resistance genes, which may modulate the responses to artemisinin combination treatments (10). This study specifically demonstrated that a mutation in codon 160 encoding a change from Ser (Ser160) to Asn/Thr (160Asn/Thr) in the P. falciparum *ap2-mu* gene was a modulator of *in vivo* responses to artemisinin derivatives in Kenyan malaria patients, being significantly more common among submicroscopic parasites surviving combination treatment at day 3 than in the pretreatment population.

To explore the role of this putative artemisinin resistance marker, we have generated transgenic P. falciparum parasites expressing an extra copy of either the wild-type (WT) *pfap2-mu* gene or the 160Asn form, driven by a heterologous promoter, in addition to the endogenous WT *pfap2-mu* gene. The susceptibility of these parasites to several antimalarial drugs (dihydroartemisinin [DHA], quinine, chloroquine, lumefantrine, mefloquine, and atovaquone) was compared to that of the parental Dd2^attB^ strain in a classical 48-h growth inhibition assay. DHA susceptibility of the parasites was further evaluated using two alternative assays. The first assay, established specifically for this study, deploys a 6-h pulse of DHA while retaining an orthodox dose-response element and does not require exhaustive parasite synchronization. This assay was compared to the recently described ring-stage survival assay (RSA), which exposes parasites at 0 to 3 h (RSA_0–3h_) postinvasion to a 6-h pulse of 700 nM dihydroartemisinin and measures survival 66 h later (11).

## MATERIALS AND METHODS

### Culture of Plasmodium falciparum.

The Dd2^attB^ clone of P. falciparum was generated by integration of the acceptor *attB* site, recognized by the mycobacteriophage Bxb1 integrase during site-specific integration, into the nonessential glutaredoxin-like *cg6* gene located on chromosome 7. Drug selection with 5 nM WR99210 was applied throughout to maintain the presence of this site, as described previously (12). Parasite cultures were maintained in complete growth medium composed of RPMI 1640 growth medium supplemented with 2.5% (vol/vol) human AB serum, 147 μM hypoxanthine, 5 g/liter Albumax II (Invitrogen), 10 mM d-glucose, and 2 mM l-glutamine at 5% hematocrit in 5% CO_2_ at 37°C. Parasite cultures were synchronized at the ring stage using sequential d-sorbitol lysis treatment (13). Where not listed, reagents were obtained from Sigma-Aldrich.

### Generation of P. falciparum transfection constructs.

**FIG 1:**
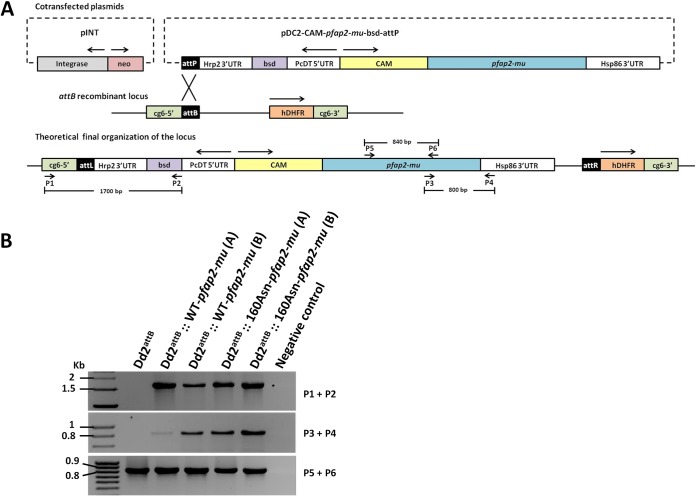
Site-specific integration of the *pfap2-mu* gene (WT and 160Asn) into the P. falciparum Dd2^attB^ line. (A) Schematic diagram of the integrase-mediated *attB* × *attP* recombination approach described by Nkrumah et al. (12). The top panel shows the cotransfected plasmids: plasmid pINT, carrying the integrase expression unit that catalyzes the recombination and the neomycin resistance cassette (neo), and the pDC2-CAM-*pfap2-mu*-bsd-attP plasmid carrying the WT or a mutant 160Asn *pfap2-mu* gene sequence under the control of the calmodulin promoter, a blasticidin resistance cassette (*bsd*) and the *attP* site. The middle panel shows the recipient *cg6-attB* recombinant locus present in Dd2^attB^. The *attB* × *attP* recombination generates two sites, *attL* (left) and *attR* (right). The human dihydrofolate reductase (hDHFR) represents the drug selection marker for WR99210. The lower panel represents the integration of the pDC2-CAM-*pfap2-mu*-bsd-attP plasmid into the *cg6-attB* locus of Dd2^attB^. The position and orientation of the PCR primers (P1 to P6) used in the analysis of the transgenic parasites are shown. (B) PCR monitoring of integration of the pDC2-CAM-*pfap2-mu*-bsd-attP plasmid on the transfected parasites (two independent transfection experiments, A and B, for the WT and the 160Asn mutant). The Dd2^attB^ DNA was used as a control. The top panel shows integration of the blasticidin cassette into the *attB* recombinant locus using the primers P1 and P2 (expected fragment size, 1,700 bp). The middle panel shows the presence of the *pfap2-mu-hsp86* 3′ UTR fusion (using the P3 and P4 primers; expected fragment size, 800 bp). The bottom panel shows the PCR product used to confirm the presence of the 160Asn mutation by sequencing (using the P5 and P6 primers; expected fragment size, 840 bp).

The open reading frames of both the wild-type and mutant (160Asn) alleles of the *pfap2-mu* gene (PlasmoDB identifier PF3D7_1218300) were amplified, respectively, from genomic DNA of P. falciparum parasite line 3D7 and from a P. falciparum field sample previously shown to harbor the 160Asn mutation (10). The PCR primers used are described in Table S1 in the supplemental material. Plasmids pDC2-*cam-pfap2-mu*-attP and the pDC2-*cam-pfap2-mu_160Asn_*-attP were then engineered (Fig. 1A). The *pfap2-mu* amplicons were cloned as AvrII/XhoI fragments between the calmodulin (PlasmoDB identifier PF3D7_1434200) promoter and the *hsp86* (PlasmoDB identifier PF3D7_0708500) 3′ untranslated region (UTR) in the pDC2 vector (14), which also encodes a blasticidin-S deaminase (*bsd*) selectable marker cassette. The resulting constructs were verified by sequence analysis, and DNA for transfection was purified using a Qiagen CompactPrep Plasmid Maxi Kit.

For each duplex transfection, 50 μg of the plasmid containing the *attP* site together with the *pfap2-mu* gene (WT or the 160Asn copy), 50 μg of the pINT plasmid containing the integrase expression unit that catalyzes the recombination, and the neomycin resistance cassette (each in 25 μl of Tris-EDTA [TE] buffer) were resuspended in 350 μl of CytoMix (120 mM KCl, 0.15 mM CaCl_2_, 2 mM EGTA [pH 7.6], 5 mM MgCl_2_, 10 mM K_2_HPO_4_/KH_2_PO_4_ [pH 7.6], and 25 mM HEPES [pH 7.6]).

### P. falciparum transfections.

Transfections of P. falciparum were carried out as described by Adjalley and colleagues (15), with slight modifications. Briefly, 250 μl of synchronized ring-stage Dd2^attB^-infected erythrocytes at 6 to 9% parasitemia was added to 400 μl of the duplex plasmid preparation in electroporation cuvettes (Bio-Rad). Parasites were transfected by electroporation (0.31 kV, 950 μF, and infinite resistance) (Gene Pulser XCell; Bio-Rad) and then immediately transferred to culture flasks containing 10 ml of complete medium and 250 μl of newly harvested red blood cells. Transfectants were allowed to recover in drug-free medium for 24 h and then selected with 2.5 μg/ml blasticidin (Sigma-Aldrich) and 125 μg/ml of G418 (Invitrogen, Life Technologies). Drug pressure with G418, selecting for the pINT plasmid, was applied for 6 days. The medium was changed daily for the first 6 days, and then every other day fresh medium containing the blasticidin and WR99210 selection agents was added. Cultures were diluted 3:5 weekly (by the addition of 30% fresh blood cells) and monitored by Giemsa staining every 4 to 5 days for the appearance of the transfected parasites. Drug pressure was maintained until the correct integration at the genomic *attB* locus was verified by PCR. Two independent transfections for each *pfap2-mu* plasmid were carried out.

Correct integration was verified by PCR using the P1/P2 primer pair (Fig. 1; see also Table S1 in the supplemental material). The presence of the *pfap2-mu* gene with the *hsp86* 3′ UTR and the *pfap2-mu* gene was also monitored using the P3/P4 and P5/P6 primer pairs, respectively (Fig. 1; see also Table S1). Sequence analysis of the mutation site G479A (160Asn) was performed using the P5/P6 primer pair.

### RNA extraction and cDNA preparation.

For total RNA isolation, parasitized red cells were rapidly lysed in TRI reagent (Sigma-Aldrich) and then stored at −80°C prior to RNA extraction, according to the manufacturer's protocol, as previously described (16). Briefly, the TRI reagent lysates were thawed at 37°C, chloroform (Sigma-Aldrich) was added, and the mixture was vigorously mixed and incubated for 15 min at room temperature before centrifugation (12,000 × *g* for 15 min at 4°C). The aqueous phase was precipitated with isopropanol (Sigma-Aldrich), incubated for 10 min at room temperature, and centrifuged (12,000 × *g* for 10 min at 4°C) to allow RNA precipitation, and the pellet was washed with 75% ethanol (Sigma-Aldrich). The samples were stored at 4°C overnight and then centrifuged at 12,000 × *g* for 10 min at 4°C. The ethanol was removed, and the RNA pellet was air dried before being resuspended in 20 μl of nuclease-free water (Promega).

Extracted RNA was treated with RQ1 RNase-free DNase (Promega) and reverse transcribed using gene-specific primers (Q2 and Q3) (see Table S2 in the supplemental material) and a GoScript reverse transcriptase kit (Promega), as described by the manufacturer. Each nuclease-treated preparation was then split in two. In the control tubes, reverse transcriptase was replaced with water (DNA contamination control).

### Quantitative RT-PCR on ring-stage and late-stage samples of P. falciparum mRNA.

Quantitative reverse transcription-PCR (RT-qPCR) was performed using a QuantiTect SYBR green PCR kit (Qiagen) in a Rotor-Gene RG3000 machine (Corbett Research). Transcripts of the *pfap2-mu* gene were amplified using the Q1/Q2 primer pair (see Table S2), and the Plasmodium tRNA methionine (PgMET) gene was used as a reference source of RNA. Previously published primer sequences Q3/Q4 (see Table S2) were used to amplify the *pgmet* gene as described previously (17). Reactions were carried out in 25-μl volumes using QuantiTect SYBR green PCR master mix according to the manufacturer's guidelines. The PCR conditions consisted of 95°C for 6 min, followed by 40 cycles of 95°C for 15 s and 60°C for 1 min for all reactions. Each parasite RNA sample was tested in triplicate in each experiment. Relative expression of *pfap2-mu* was calculated from the average threshold cycle (*C_T_*) values from two experiments normalized to *pgmet* using the ΔΔ*C_T_* method, where the parental line was used as the comparator.

### Standard 48-h drug susceptibility assays.

Standard 48-h drug exposure assays were performed to determine the susceptibility of parasites to DHA, quinine, and chloroquine (representing 4-aminoquinolines), lumefantrine and mefloquine (representing widely used ACT partner drugs), and atovaquone (representing a well-characterized antimalarial with a known mode of action independent of the lysozome) according to protocols for using the intercalating dye PicoGreen to provide a fluorescent signal (18), as previously deployed in our laboratory (19). The resulting 48-h 50% inhibitory concentration (IC_50_^48h^) estimates were used as primary indicators of antimalarial susceptibility, determined from a log dose-response analysis using Prism, version 6.04 (GraphPad Software, Inc., San Diego, CA). Each assay was performed with two replicates on at least two (range, two to six) independent occasions for each drug. For statistical analysis, best-fit estimates of the IC_50_s and their 95% confidence intervals (CIs) were obtained by nonlinear regression fitting of the sigmoidal dose-response curve for each drug across all experiments after normalization using control well fluorescent signals. For statistical comparisons between the curve of each transgenic strain and that of the parental strain and between the curves of the two transgenic strains, data were first fitted independently and then globally to find a shared best-fit value for the IC_50_^48h^. Results were compared by a sum-of-squares *F* test.

### Artemisinin 6-h pulse assays.

To generate IC_50_^6h^ estimates for artemisinin, we devised an alternative *in vitro* drug susceptibility protocol designed to reduce the exposure of parasites to artemisinin *in vitro* so as to more closely resemble the transient drug exposure of ACT-treated parasites *in vivo*. DHA was serially diluted in complete medium (250 μl) in Eppendorf tubes, using the same 10 concentrations generated for the standard 48-h drug assays, so as to generate a full dose-response curve. A total of 250 μl of ring-stage culture, synchronized by a single round of 2% d-sorbitol treatment (0 to 12 h postinvasion), was added to each drug dilution (2% final hematocrit and 0.5% final parasitemia) and incubated at 37°C for 6 h. After the incubation time, half of the culture was placed in a 96-well tissue culture plate (modified 48-h assay), and the other half was washed three times with 1,000 μl of RPMI medium to remove DHA, before being replenished with drug-free medium and placed in a 96-well culture plate. The plate was then incubated at 37°C for 42 h until growth assessment using the PicoGreen detection method, as described for the standard 48-h assays, generating a measure of DHA sensitivity in the form of an IC_50_^6h^ estimate.

### Ring-stage survival assay (RSA_0–3h_).

The RSA_0–3h_ was performed as described by Witkowski and colleagues (11) with minor modifications. The parasite cultures were tightly synchronized across two consecutive ring-stage cycles with sorbitol treatment. Late-schizont-stage parasite cultures were enriched using magnetic cell sorting (MACS) separation columns (Miltenyi Biotech GmbH) and were cultured for 3 h at 37°C with fresh erythrocytes and again sorbitol treated. This early-ring-stage (0 to 3 h) parasite preparation, at 1% parasitemia and 2% hematocrit in a 2-ml final volume, was then exposed for 6 h to 700 nM DHA in 0.1% dimethyl sulfoxide (DMSO). After the 6-h exposure the cultures were washed and resuspended in drug-free culture medium and cultured at 37°C for a further 66 h. Dihydroartemisinin susceptibility was then assessed microscopically on thin films by estimating the percentage of viable parasites that had developed into a new generation of trophozoites 66 h after DHA exposure compared to results for parasites exposed to 0.1% DMSO alone. This assay does not generate a dose-response curve.

## RESULTS

### Generation of transgenic parasites by integration of an additional copy of *pfap2-mu* in Dd2^attB^.

To investigate the potential role for the *pfap2-mu* 160Asn mutation in mediating reduced *in vitro* susceptibility of P. falciparum to antimalarial drugs, we generated transgenic parasites with an additional copy of either the wild-type Ser160 or the 160Asn mutant form of the *pfap2-mu* gene. This was achieved using Bxb1 mycobacteriophage integrase-mediated recombination to deliver transgenes into the P. falciparum genome in a site-specific manner, dictated by the presence in the parental line Dd2^attB^ of the *attB* target sequence, replacing the *cg6* locus (12). The *pfap2-mu* gene, with or without the 160Asn mutation, was cloned into the pDC2 plasmid under the control of the calmodulin promoter (Fig. 1A) and cotransfected into the Dd2^attB^ strain with the pINT plasmid (encoding integrase) (12). After transfection the parasites were placed under drug selection with both blasticidin and G418, generating two transgenic lines, termed Dd2^attB^::WT-*pfap2-mu* and Dd2^attB^::160Asn-*pfap2-mu*. Stably transfected parasites were obtained 2 to 3 weeks after transfection, and diagnostic PCR using P1/P2 specific primers (Fig. 1 A; see also Table S1 in the supplemental material) revealed correct integration into the *attB* site. The presence of the *pfap2-mu* transgenes was confirmed by PCR analysis (Fig. 1 B; see also Table S1), and the presence of the 160Asn mutation in the Dd2^attB^::160Asn-*pfap2-mu* line was confirmed by sequencing.

### RT-qPCR analysis of *pfap2-mu* in transgenic lines.

**FIG 2:**
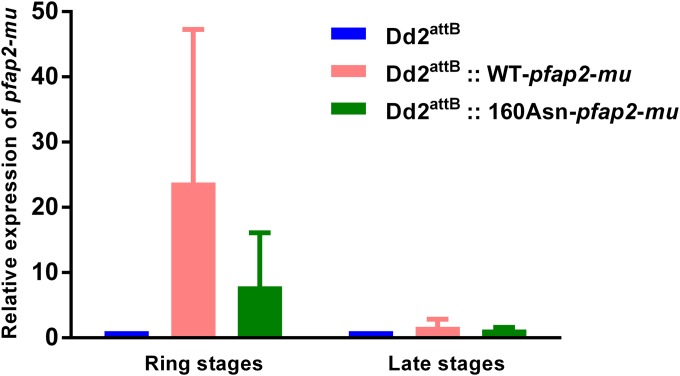
Expression levels of *pfap2-mu* transcripts on ring-stage and late-stage (trophozoites and schizonts) cultures of Dd2^attB^, Dd2^attB^::WT-*pfap2-mu*, and Dd2^attB^::160Asn-*pfap2-mu* lines. Mean mRNA expression levels of *pfap2-mu* in Dd2^attB^ and transfected lines from RT-qPCR analysis are shown. The expression of *pfap2-mu* was normalized to the mRNA level of PgMET. Error bars indicate standard deviations.

RT-qPCR analysis of cultures of the parental and transfected parasite lines provided evidence of constitutive blood-stage expression of the introduced transgenes. In ring-stage cultures, 7.9-fold (160Asn-*pfap2-mu*) and 23.8-fold (WT-*pfap2-mu*) overexpression of *pfap2-mu* mRNA compared to the level in the parental line was found in the transfectant lines, reflecting the known expression profile of the calmodulin promoter (highly active across the 48-h cycle with a distinct peak during the mid-trophozoite stage), but late-stage cultures of both transfectant lines exhibited mRNA levels similar to those of Dd2^attB^ (Fig. 2). For each sample, parallel reactions performed without reverse transcriptase did not generate detectable amplicons within 35 cycles, demonstrating the absence of detectable genomic DNA contaminating the RNA (data not shown). Total RNA prepared from the peripheral blood of a malaria patient as part of a previous study (19) was also analyzed and confirmed transcription of *pfap2-mu* by circulating P. falciparum blood-stage parasites *in vivo*.

### *In vitro* sensitivity of *pfap2-mu* transfectant parasites.

**FIG 3:**
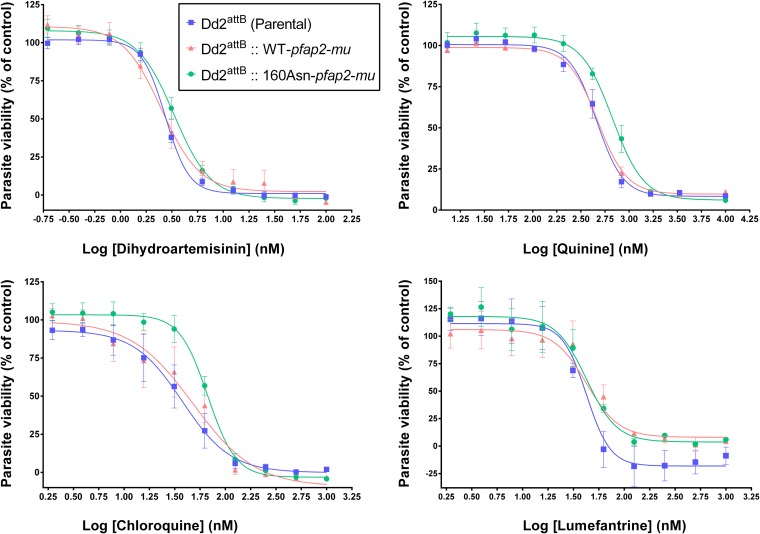
Dose-response curves of the Dd2^attB^ (parental), Dd2^attB^::WT-*pfap2-mu*, and Dd2^attB^::160Asn-*pfap2-mu* strains for dihydroartemisinin, quinine, chloroquine, and lumefantrine. The drug sensitivity of the parasites was assessed using a standard 48-h assay. Each point represents the mean of at least two independent experiments, with two replicates for each experiment. The error bars indicate ± standard errors of the means. Best-fit curves were generated by Prism, version 6.04. The *x* axis indicates the increasing concentrations of the different drugs, and the *y* axis indicates the parasite viability.

The growth inhibition curves for DHA, quinine, chloroquine, and lumefantrine for both transgenic lines are presented in Fig. 3. Table 1 shows the mean (± standard deviation) IC_50_^48h^ estimates calculated for the parental and transfected lines and the significance of any differences in sensitivity between the different lines. In four independent experiments, the transgenic parasite line harboring the 160Asn copy of the *pfap2-mu* gene displayed a significantly reduced sensitivity to DHA (mean IC_50_^48h^, 3.3 nM) compared with the sensitivities of both the parent line (mean IC_50_^48h^, 2.7 nM) and WT-*pfap2-mu* (mean IC_50_^48h^, 2.5 nM) lines (Table 1). The quinine IC_50_^48h^ values increased significantly from 459.2 nM in the parental line and 471.5 nM in the WT-*pfap2-mu* to 671.2 nM in the 160Asn-*pfap2-mu* parasite line. The presence of 160Asn-*pfap2-mu* appeared to reduce susceptibility to chloroquine compared with that of the parental line. However, the WT-transfected line exhibited an intermediate phenotype which was not statistically different from the phenotypes of either the parental or mutant transgenic lines, and so no definite conclusions can be made from the current data. Thus, decreased susceptibility to DHA and quinine was conferred in these experiments by the presence of the 160Asn *pfap2-mu* mutant allele, given that the overexpression of the WT allele had no effect on parasite susceptibility to these antimalarials. In contrast, the Dd2^attB^::WT-*pfap2-mu* line showed significantly reduced susceptibility to lumefantrine (IC_50_ of 57.7 nM) compared with that of the parental line (IC_50_ of 36.1 nM). However, as no significant difference was found between the WT and the 160Asn lines or between the 160Asn and the parental lines, the importance of this observation remains unclear. The presence of 160Asn-*pfap2-mu* did not significantly alter parasite susceptibility to atovaquone, a drug with a well-described mitochondrial mode of action that is unrelated to that of the artemisinins or aminoquinolines, nor did it alter mefloquine IC_50_s (Table 1).

**TABLE 1:** *In vitro* drug susceptibility of the Dd2^attB^ (parental), Dd2^attB^::WT-*pfap2-mu*, and Dd2^attB^::160Asn-*pfap2-mu* transfectant strains to six antimalarial drugs

Drug (*n*)^*a*^	IC_50_ (nM) (95% CI)^*b*^			*P* value^*c*^		
	Dd2^attB^ (parental)	Dd2^attB^::WT-*pfap2-mu*	Dd2^attB^::160Asn-*pfap2-mu*	WT vs Dd2^attB^	160Asn vs Dd2^attB^	WT vs 160Asn
Dihydroartemisinin (4)	2.7 (2.6–2.9)	2.5 (2.0–3.1)	3.3 (2.9–3.8)	0.371	**0.0057**	**0.0227**
Quinine (3)	459.2 (413.3–510.3)	471.5 (442.1–502.9)	671.2 (582.3–773.6)	0.683	**<0.0001**	**<0.0001**
Chloroquine (4)	37.6 (25.9–54.5)	48.0 (29.6–78.0)	67.1 (58.6–76.8)^*d*^	0.445	**0.0028**	0.1429
Lumefantrine (2)	36.1 (28.3–46.2)	57.7 (43.0–77.4)	44.8 (32.3–62.1)	**0.0294**	0.3028	0.2517
Mefloquine (6)	13.1 (10.3–16.7)	16.7 (13.9–19.9)	17.0 (13.6–21.2)	0.1416	0.1488	0.8829
Atovaquone (4)	2.7 (2.3–3.4)	2.8 (2.3–3.5)	4.0 (2.7–6.0)	0.923	0.1014	0.1127

a *n*, number of independent experiments. Each experiment had two replicates.

b The drug sensitivity of the parasites was assessed using a classic 48-h growth inhibition assay. The best-fit curve for each drug was generated in Prism, version 6.04, and the best-fit estimate of the IC_50_s and their 95% confidence intervals (CIs) are indicated.

c A sum-of-squares *F* test was used to test the significance of difference among the IC_50_s of the different parasites. The significant *P* values are indicated in bold.

d For Dd2^attB^::160Asn-*pfap2-mu*, only 3 experiments with chloroquine were performed.

**FIG 4:**
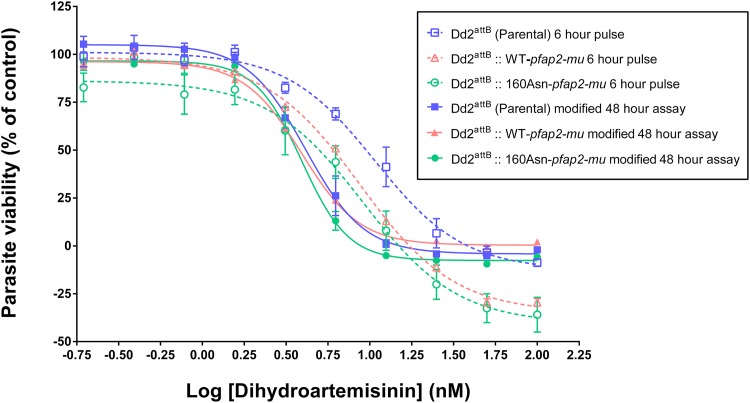
Dose-response curves of the Dd2^attB^ (parental), Dd2^attB^::WT-*pfap2-mu*, and Dd2^attB^::160Asn-*pfap2-mu* transfectants for dihydroartemisinin. The drug sensitivity of the parasites was assessed using a modified 48-h assay (solid line) and 6-h pulse assay (dashed line). Each point represents the mean of two independent experiments, with two replicates for each experiment. The error bars indicate ± standard errors of the means. Best-fit curves were generated by Prism, version 6.04. The *x* axis indicates the increasing concentration of dihydroartemisinin, and the *y* axis indicates the parasite viability.

The 6-h drug pulse assay, developed to better reflect the short exposure times to artemisinin that occur *in vivo*, was used to estimate the DHA IC_50_^6h^ for parental and transgenic lines. Figure 4 shows the best-fit curves generated, and IC_50_^6h^ estimates are indicated in Table 2. The survival curves obtained in the 6-h pulse assay showed a rightward shift toward higher DHA concentrations than the survival curves obtained in the modified 48-h assay (i.e., with a 6-h tube incubation prior to plating) (see Materials and Methods). These curve shifts meant that DHA IC_50_^6h^ estimates were always higher than the IC_50_^48h^ values by approximately 2-fold. Nevertheless, no significant difference was found between the transfectant and the parental lines using either the IC_50_^48h^ or IC_50_^6h^ estimate (Table 2). The IC_50_s obtained in the modified 48-h assay were slightly higher (range, 3.9 to 4.1 nM) than the ones obtained in the standard 48-h assay (range, 2.5 to 3.3 nM), possibly due to differences in drug exposure arising from the initial 6-h incubation in microcentrifuge tubes.

**TABLE 2:** *In vitro* drug susceptibility of the Dd2^attB^ (parental), Dd2^attB^::WT-*pfap2-mu*, and Dd2^attB^::160Asn-*pfap2-mu* transfectant strains to dihydroartemisinin using a 6-h pulse assay

Strain	IC_50_ (nM) (95% CI)^*a*^	
	Modified 48-h assay	6-h pulse assay
Dd2^attB^ (parental)	4.1 (3.7–4.6)	10.8 (8.6–13.5)
Dd2^attB^::WT-*pfap2-mu*	3.9 (3.8–4.1)	8.8 (8.1–9.4)
Dd2^attB^::160Asn-*pfap2-mu*	3.9 (3.6–4.2)	9.1 (6.3–13.1)

a The dihydroartemisinin susceptibility of each parasite line was assessed in both assays by two independent experiments, with two replicates for each experiment.

The susceptibility of young ring-stage (0 to 3 h) cultures to a 6-h pulse with 700 nM DHA was evaluated using the RSA_0–3h_ of Witkowski et al. (11). Parasite viability was zero, defined as more than 99% of parasites recorded as dead at 72 h, in all test wells (data not shown), indicating that parental and transgenic lines were fully susceptible to DHA by this test.

## DISCUSSION

In this study, we aimed to better understand the role of the *ap2-mu* gene in influencing P. falciparum drug responses. *pfap2-mu* was first implicated in artemisinin resistance in the rodent malaria parasite P. chabaudi through whole-genome sequencing of a parasite lineage resistant to artemisinin, which identified a novel Ile568Thr change as responsible for this phenotype by genome-wide association analysis (8). Several variants of this gene were identified in P. falciparum, but a polymorphism homologous to P. chabaudi Ile568Thr has not been detected in field isolates to date (8, 10). However, our recent work conducted in Kenya provides evidence that a Ser160Asn/Thr mutation can modulate the parasite response to artemisinin combination treatments *in vivo* (10). Consistent with these results, we now show that transgenic parasites carrying 160Asn *pfap2-mu* are significantly less susceptible to DHA *in vitro*, manifest as a 32% increase in the DHA IC_50_ compared to the level in transgenic parasites carrying an additional copy of the wild-type *pfap2-mu* locus, using a standard 48-h drug susceptibility testing protocol (18, 19). Unexpectedly, transgenic parasites carrying the 160Asn *pfap2-mu* allele were much less susceptible to quinine than were those with the wild-type allele, raising the intriguing possibility that quinine pressure in Kenya may have been responsible for selecting the 160Asn allele in the parasite population (10). In addition, this study provides weak evidence that changes in the *pfap2-mu* sequence (for chloroquine) or expression level (for lumefantrine) also affect parasite sensitivity to other antimalarials *in vitro*. Further experiments are needed to confirm that the higher early-stage mRNA expression measured here is accompanied by increased expression of the corresponding protein but await the development of suitable reagents (i.e., specific antibodies). Additionally, it will be important to compare *in vitro* susceptibility of the transgenic parasites to that of Cambodian parasite lines with known clearance time phenotypes in both the 48-h exposure dose-response assessment, generating IC_50_ estimates, and in the RSA_0–3h_ assay.

The *pfap2-mu* gene is predicted to encode the mu (μ) subunit of the adaptor protein 2 (AP2) complex. AP2 facilitates formation of clathrin-coated vesicles for the trafficking of cargo from one membrane compartment of the cell to another by recruiting a number of other proteins to the forming vesicle (20). AP2 is located at the parasite plasma membrane and also contributes to the selection of specific cargo (21). The mu subunit binds to the cytoplasmic side of cargo molecules through recognition of specific signals and is incorporated into the mature clathrin-coated vesicle (22), suggesting that AP2-mu variants may influence drug resistance by modulating cargo trafficking (8). The precise mechanism by which polymorphisms in *pfap2-mu* affect antimalarial drug response remains unknown. The P. chabaudi Ile568Thr mutation is predicted to lie in the C terminus of the AP2-mu protein, whereas the Ser160Asn mutation in P. falciparum is positioned in the N-terminal domain. Both regions are highly conserved across the genus, and by analogy to studies of mammalian homologues, it is predicted that both codons contribute to β-sheet structure lying within or adjacent to the hydrophobic pocket that binds to the recognition motif present in the cargo protein (7). These mutations may therefore mediate the antimalarial drug response by reducing AP2-mu binding affinity to the membrane cargo and thus decreasing the efficiency of endocytotic trafficking of membrane proteins. A better understanding of AP2 variants requires studies of the structure and function of the adaptor complex protein in Plasmodium spp.

The phenotype of reduced susceptibility to DHA described in this study is moderate and unlikely to confer drug resistance in itself; the phenomenon also appears to be significantly different from the slow-clearance phenotype described in Southeast Asia, which is associated with mutations in the *pfk13* kelch propeller domain (6, 23). First, the latter is associated with prolonged parasite clearance times *in vivo* and with increased survival in the ring-stage parasite survival assay but not with any change in *in vitro* susceptibility to DHA using a standard 48-h drug assay. Here, the transgenic population carrying the mutant *pfap2-mu* was shown to be significantly less susceptible to DHA than the parental population even though the IC_50_ was only marginally increased; however, this “tolerant” phenotype was not detected using the RSA_0–3h_ and is thus distinct from the phenotype described by Witkowski et al. (11). The three assays we deployed are not measuring the same phenotypes as the duration of drug exposure differs markedly, and the RSA assay has no dose-response element. Second, in contrast to the Southeast Asian isolates, the persistent Kenyan parasites carrying the Ser160Asn/Thr mutation also carry the chloroquine-sensitive haplotype *pfcrt* CVMNK and were microscopically undetectable; to date the *pfk13* genotypes associated with the Southeast Asian reduction in susceptibility have not been seen in Kenyan parasites represented by the currently available whole-genome sequence data (10).

The modulation of artemisinin sensitivity in an African context clearly manifests as a different phenotype from the one described in Cambodia, with different genes and genotypes implicated; for example, no *pfap2-mu* codon 160 polymorphisms are seen in publicly available genomes of any Southeast Asian isolates (10). Conversely, African slow-clearing infections are not associated with polymorphisms in the kelch protein (4), suggesting the contribution of other genetic factors. Furthermore, drug resistance may not arise in a single step but as a long and complex process during which parasites become gradually more and more tolerant to the drug. This might result from the stepwise accumulation of genetic changes in the same gene or in different genes (24). Therefore, it is possible that mutations in AP2-mu are an early step in one possible pathway for the development of artemisinin resistance and that additional genetic changes would be required before a fully resistant parasite genotype has emerged. This is supported by our recent study in Kenya where variants at the *pfcrt*, *pfmdr1*, and *pfubp1* loci, as well as the *pfap2-mu* mutations at codon 160, were associated with posttreatment parasite persistence (10). No genetic similarities to Cambodian P. falciparum were seen at the four loci evaluated in this study, suggesting that the two *in vivo* phenotypes are not related. Alternatively, AP2-mu may contribute to a broad parasite heterogeneity that modulates the drug response to several antimalarial drugs, as seen for *pfmdr1* and other members of the ABC transporter family in P. falciparum (25).

A potential restriction of our study is the sole use, as the parent isolate, of Dd2^attB^, a transgenic line derived from the multidrug-resistant Dd2 line that carries a chloroquine-resistant *pfcrt* allele and a multicopy *pfmdr1* locus conferring reduced mefloquine susceptibility but that remains sensitive to artemisinin. Dd2^attB^ was chosen for this purpose because in our hands it propagates more rapidly than the few other lines available harboring the *attB* site (14). The genetic background of the parasite can influence the acquisition of drug resistance, so the drug responses observed in this study may vary in isolates with a different preexisting drug sensitivity background. For example, a significant increase in the lumefantrine IC_50_^48h^ to 57.7 nM was observed in the Dd2^attB^::WT-*pfap2-mu* transgenic parasites, which is well within the range we have observed in patient isolates tested *ex vivo* (17). However, the highest IC_50_^48h^ among these patient isolates occurred in those isolates that were wild type for the chloroquine-associated resistance marker *pfcrt*. In order to better understand the role of AP2-mu in drug resistance, it is important to perform further genetic studies in isolates with different backgrounds, including those with chloroquine-sensitive genotypes and those with *pfk13* mutations, and we are now pursuing such studies with recently developed gene-editing approaches (26). Recent studies have now confirmed the association between the artemisinin response *in vitro* and polymorphism in *pfk13* by demonstrating an increase in survival in the RSA_0–3h_ assay in parasites engineered to express K13 mutations, as well as a loss of resistance in parasites whose mutant K13 gene was reverted back to the wild-type sequence (23).

In summary, this study shows that expression of 160Asn *pfap2-mu* in Dd2^attB^ altered P. falciparum susceptibility to DHA, with transgenic parasites exhibiting significantly higher IC_50_^48h^ estimates. This confirms *pfap2-mu* as a locus of interest for studies of artemisinin susceptibility. Our data also provide evidence that *pfap2-mu* variants can modulate parasite susceptibility to quinine *in vitro*.

## Supplementary Material

Supplemental material
