# Supplementary material for: The Mu Subunit of Plasmodium falciparum Clathrin-Associated Adaptor Protein 2 Modulates In Vitro Parasite Response to Artemisinin and Quinine
Source: Antimicrob Agents Chemother. 2015 Apr 10;59(5):2540–7. doi: 10.1128/AAC.04067-14 (PMC4394773; doi:10.1128/AAC.04067-14)
Supplement: Supplemental material [file supp_59_5_2540__index.html]

The Mu Subunit of Plasmodium falciparum Clathrin-Associated Adaptor Protein 2 Modulates In Vitro Parasite Response to Artemisinin and Quinine — Supplemental material 

# The Mu Subunit of Plasmodium falciparum Clathrin-Associated Adaptor Protein 2 Modulates *In Vitro* Parasite Response to Artemisinin and Quinine

## Supplemental material

**Files in this Data Supplement:**

- Supplemental file 1 -

  Supplemental Table S1: primer sequences used in vector construction and integration confirmation. Table S2: primer sequences used in RT-qPCR on *P. falciparum* mRNA.

  PDF, 415K
