# Supplementary material for: The Mu Subunit of Plasmodium falciparum Clathrin-Associated Adaptor Protein 2 Modulates In Vitro Parasite Response to Artemisinin and Quinine
Source: Antimicrob Agents Chemother. 2015 Apr 10;59(5):2540–7. doi: 10.1128/AAC.04067-14 (PMC4394773; doi:10.1128/AAC.04067-14)
Supplement: Supplemental material [file AAC.04067-14_zac005153896so1.pdf]

**Table S1.** Primer sequences used in vector construction and integration confirmation

| Primer | Sequence (5' – 3') | Description |
|---|---|---|
| C1 | CGTAACTAGTATGATCGATGCGCTGTACAT | Amplification of the *pfap2-mu* ORF (Forward) |
| C2 | ATGCCTCGAGCTATTTATACTGGTAGATGCC | Amplification of the *pfap2-mu* ORF (Reverse) |
| P1 | CCAGGATCCAAAAGAAGGAGGAGG | Confirming integration of bsd cassette into the *attB* recombinant locus (Forward) |
| P2 | ATGCATGCCAAGCCTTTGTCTCAAG | Confirming integration of the bsd cassette into the *attB* recombinant locus (Reverse) |
| P3 | GCATATTTCATCATTGTGTTACC | Confirming the presence of the *pfap2-mu*-Hsp86 3'UTR fusion (Forward) |
| P4 | GGGGTGATGATAAAATGAAAGATA | Confirming the presence of the *pfap2-mu*-Hsp86 3'UTR fusion (Reverse) |
| P5 | GATATCCACAAACATTAGAAGTG | Confirming the presence of the *pfap2-mu* gene (Forward) |
| P6 | CCATCTGGTGGTGTGAAGG | Confirming the presence of the *pfap2-mu* gene (Reverse) |

**Table S2.** Primer sequences used in RT-qPCR on *P. falciparum* mRNA

| Primer | Sequence (5' – 3') | Description |
|---|---|---|
| Q1 | GGAAAGGTAACCTTAAAATGTC | *pfap2-mu* amplification (Forward) |
| Q2 | GTTACTACCGGCGAGAATATT | *pfap2-mu* amplification (Reverse) and cDNA synthesis |
| Q3 | TGAAAGCAGCGTAGCTCAGA | tRNA amplification (Forward) |
| Q4 | CGCGTGGTTTCGATCCACG | tRNA amplification (Reverse) and cDNA synthesis |
